# Essential role of inverted repeat in Epstein–Barr virus IR-1 in B cell transformation; geographical variation of the viral genome

**DOI:** 10.1098/rstb.2018.0299

**Published:** 2019-04-08

**Authors:** Ray Bridges, Samantha Correia, Fanny Wegner, Cristina Venturini, Anne Palser, Robert E. White, Paul Kellam, Judith Breuer, Paul J. Farrell

**Affiliations:** 1 Section of Virology, Faculty of Medicine, Imperial College London, London W2 1PG, UK; 2 Division of Infection and Immunity, University College London, Gower Street, London WC1E 6BT, UK; 3 Wellcome Trust Sanger Institute, Hinxton, Cambridge CB10 1SJ, UK

**Keywords:** Epstein–Barr virus, strain variation, repeats, cell transformation

## Abstract

Many regions of the Epstein–Barr virus (EBV) genome, repeated and unique sequences, contribute to the geographical variation observed between strains. Here we use a large alignment of curated EBV genome sequences to identify major sites of variation in the genome of type 1 EBV strains; the CAO deletion in latent membrane protein 1 (LMP1) is the most frequent major indel present in the unique regions of EBV strains from various parts of the world. Principal component analysis was used to identify patterns of sequence variation and nucleotide positions in the sequences that can distinguish EBV from some different geographical regions. Viral genome sequence variation also affects interpretation of genetic content; known genes, origins of replication and gene expression control regions explain most of the viral genome but there are still a few sections of unknown function. One of these EBV genome regions contains a large inverted repeat sequence (invR) within the IR-1 major internal repeat array. We deleted this invR sequence and showed that this abolished the ability of the virus to transform human B cells into lymphoblastoid cell lines.

This article is part of the theme issue ‘Silent cancer agents: multi-disciplinary modelling of human DNA oncoviruses’.

## 1. Introduction

Most of the genetic map of Epstein–Barr virus (EBV) is occupied by known genes or other genetic elements, whose functions have been demonstrated experimentally (annotated in the reference EBV genome sequence NC007605). The approximately 172 kb genome of EBV includes many different tandem repeated sequences. Some of these are within the open reading frames and are expressed as sections of repetitive protein structure within certain EBV proteins. Some other repeat regions are not in open reading frames but have specific functions [1]. The largest repeat (IR-1 or major internal repeat) comprises about 6.6 copies of a 3072 nt sequence; it includes the Wp promoter and some exons of the Epstein-Barr nuclear antigen (EBNA) mRNAs. These, however, only account for about half the IR-1 repeat sequence and IR-1 also contains a large open reading frame (BWRF1). No mRNA has yet been found for BWRF1 [2]. The BWRF1 open reading frame and an inverted repeat sequence (invR) within it are both conserved in EBV strains [3].

Our recent studies have provided Epstein–Barr virus (EBV) genome sequences from a wide range of worldwide geographical locations, both from diseases associated with EBV and from healthy infected people [4–6]. There are large variations in the incidence of some EBV-associated diseases in different parts of the world. The incidence of EBV-associated nasopharyngeal carcinoma (NPC) is about 50-fold higher in Cantonese parts of Southern China than in western countries; EBV-associated Burkitt's lymphoma (BL) is frequent in sub-Saharan Africa and natural killer/T cell lymphomas containing EBV are relatively frequent in some Asian countries. In contrast, infectious mononucleosis (owing to delayed primary infection by EBV) is common in Western countries [1]. Host genetic risk, behavioural/lifestyle factors, co-infections and environmental carcinogens account for some of the difference in incidence but differences in EBV strains endemic in different parts of the world may also contribute to disease incidence [1]. Natural variation of the gp350 and gp42 EBV glycoproteins [6], which are the antigens being used in current efforts to make an EBV vaccine, may also be relevant to the universal application of the vaccine.

Although a phylogenetic tree is an effective way of analysing viral genome sequence relationships (for example fig. S1 of [6]), there are theoretical difficulties in using that method with long sequences that diversify through inter-strain recombination [7] and there is also no direct way to deduce which nucleotide positions determine the different branches of the phylogeny. We have previously reported the extensive historical recombination apparent in EBV strain sequences [4]. We used principal component analysis (PCA) to identify regions of the genome that correspond to major components of variation [4,6]. The type 1/type 2 difference is the major form of variation and the sequences within the EBV genome that determine the differences between type 1 and type 2 EBV have been described previously using this dataset [6].

In this paper we analyse the geographical variation between EBV strains, using PCA to identify individual nucleotides in the viral genome that are sufficient to distinguish some of the main patterns of geographical variation of viral strains. The large repeat arrays in the EBV genome remain a challenge for sequence analysis because the standard Illumina sequence reads are not long enough to span the repeat units. Alternative methods that give longer sequence reads (PacBio, Oxford Nanopore) can be used on cloned viral DNA but are not suitable for the very low fraction of viral DNA in clinical samples or EBV infected cell lines. The hybrid enrichment protocols that have been used to increase sequencing efficiency [4,6] involve fragmenting the DNA, again limiting the length of sequence reads. However, progress has been made in de-convoluting the IR-1 repeat region [3] and here we use our recently improved genetic method for modifying EBV repeats [8] to show the essential requirement for a large inverted repeat sequence (invR) present in each copy of IR-1 for transformation of human B cells by EBV.

## 2. Methods

The 241 EBV genome sequences [6] studied here (listed in electronic supplementary material, table S1) were aligned using MAFFT [9], inserting gaps (shown as – in the sequences) to achieve the multiple sequence alignment (MSA). Some manual editing was used to reduce gapping in repeat sequence alignments. The prototypic reference EBV sequence is Accession Number NC007605, which is a chimaera of B95-8 and Raji EBV. The gapping in the MSA means that the MSA has a length of 178 176 nt compared to the 171 823 nt of NC007605.

Only the 217 type 1 EBV genomes from the MSA were used in the analysis of geographical variation shown in this paper. Of the 178 176 nucleotide locations in the MSA, 7708 were found to have a single nucleotide polymorphism (compared to the consensus) in five or more genomes. This provided a 7708 × 217 matrix of SNP locations as the basis for our search for those nucleotides that could be associated with the geographical variation observed. The PCA technique adopted was performed using a singular value decomposition procedure from the Delphi/Pascal-based SDL Component Suite (http://www.lohninger.com). An initial transformation of the matrix enabled the 7708 eigenvector coefficients to be derived in less than a minute on a desktop computer. As before [6], the PCA in this paper counts gaps in the MSA in addition to the usual single nucleotide polymorphisms (SNPs).

Construction of the invR deletion mutant of the B95-8 EBV bacterial artificial chromosome (BAC) used a procedure analogous to our recent study of EBNA-LP [8] to introduce the deletion into all copies of IR-1. Briefly, the invR nucleotides 15 407–15 917 (EBV NC007605 numbering) were replaced by a 15 bp linker containing restriction sites in a single BamHI fragment of IR-1 (and in the first partial IR-1 repeat upstream of the first BamHI site) by PCR. Gibson assembly was used to construct a repeat array of 6.6 tandem copies of this mutated IR-1 repeat that was recombined into the W4 EBV BAC lacking IR-1 as described previously [8], generating invR-deleted EBV. To construct a revertant, the invR-deleted EBV IR-1 region was deleted by recombineering and replaced with a wild-type repeat array. Full details of the sequences used, procedures and verification of the recombined viruses are available online [10]. The B cell transformation assay using indicated green Raji units (GRU) of virus [11] with 10^6^ B cells per ml and western blot for EBNA-LP using antibody JF186 [12] were as described [8,10].

## 3. Results

### (a) Multiple sequence alignment file

**Figure 1.**
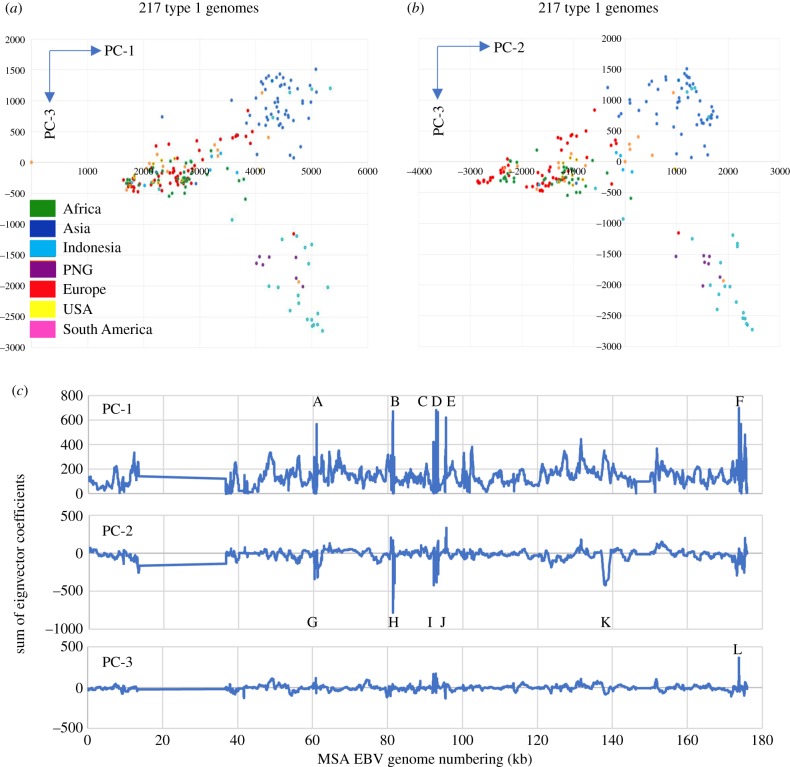
Principal component analysis of type 1 EBV sequence diversity coloured by geographical origin (colours as shown in part *a*) using 217 type 1 EBV strains (*a*) PC-1 versus PC-3, (*b*) PC-2 versus PC-3. PC-1 versus PC-2 with the same dataset is shown in fig. 1B of [6]. (*c*) Eigenvector coefficients plotted with a smoothing window of 20 nt along the MSA genome (plotted as sum of coefficients on the ordinate axis). Labelled peaks are listed in electronic supplementary material, figure S2. PNG, Papua New Guinea.

We recently reported [6] some preliminary analysis of a multiple sequence alignment (MSA) of a total of 241 EBV genomes. This 43 Mb MSA file is now provided as electronic supplementary material, figure S1 in fasta format; it forms the basis of the EBV sequence analysis described in this paper and in [6]. The details of all the strains in the MSA including GenBank accession numbers have been described previously [6]; electronic supplementary material, table S1 lists the standard strain names [6] together with the decorated names of EBV strains used in electronic supplementary material, figure S1 that allow representation of the geographical origin and other features in the PCA. The first three components of variation in the 241 sequences in the MSA account for 45% of the variation and readily separated the type 1 from the type 2 sequences [6]. PC1 was 26.9%, PC2 was 12.1% and PC3 was 6.1% of the variation. Geographical separation of strains is also apparent in these PCA results ([6] and figure 1*a*,*b*). In this conventional presentation of the PCA (figure 1*a*,*b*), each point in the figure corresponds to one EBV genome sequence. The position of the point corresponds to the sum of the eigenvector coefficients for those positions in the sequence that have a SNP relative to the consensus sequence.

### (b) Major indels in the BART miRNA and LMP1 regions

In an initial approach to find genome sequences that might determine geographical clustering, we had identified some geographical groups of type 1 strains which clustered closely in a phylogenetic tree [6] and PCA (figure 1*a*,*b*), which were considered characteristic of a geographical region. These groups of isolates from Indonesia, Papua New Guinea, Africa (Kenya) and China were relatively homogeneous and comparison of SNPs between their consensus sequences and a consensus of all 217 type 1 sequences provides one approach to identifying the variation (electronic supplementary material, figure S2). To focus on the main points of variation, nucleotide locations were only included in these analyses when the number of occurrences of SNPs at the location in question was greater than 5. The number of SNPs at each of these greater than 5 locations was plotted for each group separately as cumulative SNPs in a 100 nt window and this identified regions and peaks of difference (electronic supplementary material, figure S2). Many of these peaks were at similar genome locations in several of the geographical groups and were in repeat arrays but there were some differences. The gene regions corresponding to the electronic supplementary material, figure S2 peaks are listed in electronic supplementary material, table S2. We previously reported [6] the insertion in the BART region of the genome (electronic supplementary material, table S2), between miR BART8 and miR BART21, in 21 of the 241 strains analysed.

**Figure 2.**
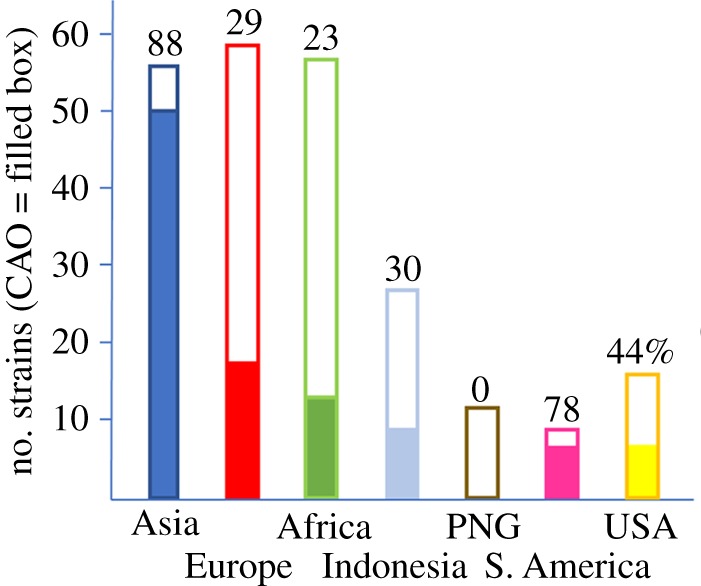
CAO deletion incidence in EBV strains from different geographical regions. The filled part of the boxes indicates the number of CAO strains within the total, also expressed as a percentage for each strain. PNG, Papua New Guinea.

In fact, the most frequent major indel detected by this type of analysis (peak 167798 in electronic supplementary material, figure S2) is a 30 nt region of the *LMP1* gene and is known as the CAO deletion. It was originally identified in a Chinese nasopharyngeal carcinoma sample [13] and its functional consequences for LMP1 have been studied extensively, summarized in [14]. LMP1 with this deletion is more effective at activating NF-kB [14] and is more tumorigenic in rodent fibroblasts or epithelial cells implanted into nude mice [15–17]. The 30 bp CAO deletion is very uniform in those strains that have it (see nucleotide positions 173 802–173 831 in electronic supplementary material, figure S1) and we can now estimate its varying prevalence in different geographical regions (figure 2). Overall, it was present in 99 of 232 strains (43%) that were analysed here and had a high incidence in the Asian and South American strains we studied.

### (c) Distribution of geographical variation in the Epstein–Barr virus genome

The presentation of results in electronic supplementary material, figure S2, plotting number of SNPs in a sliding window, tends to accentuate regions where many SNPs are clustered together and thus mostly picked out repeat arrays or larger indels (electronic supplementary material, table S2). Plotting the coefficients for each of the first three eigenvectors (figure 1*c*) showed the broad regions of the EBV genome that contribute to the distributions in the PCA and the locations of those main peaks are also listed in electronic supplementary material, table S2, with some details of their corresponding EBV genome region.

**Figure 3.**
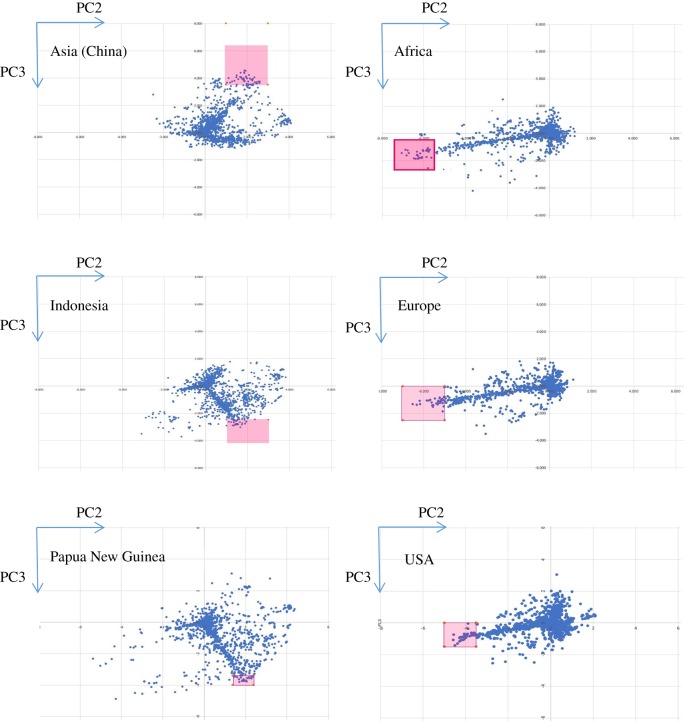
PCA nucleotide contribution plots of geographical groups identify three major patterns consistent with three major clades in the phylogenetic tree of type 1 sequences. Nucleotide positions from the marked areas, which were chosen to characterize differences between the geographical groups, are listed in electronic supplementary material, table S3.

To focus more specifically on some single SNPs that contribute strongly to the geographical separation observed in the PCA plots and phylogenetic tree (figure 1 and [6]), we plotted the PC2 and PC3 coefficients of all the nucleotide positions that contribute to the PCA of geographical groups of EBV strains. In this alternative presentation of the PCA, the eigenvector coefficients for all sequences in the chosen group are averaged at each of the 7708 positions in the EBV genome that have a SNP and thus contribute to the PCA. Each point in figure 3 thus corresponds to one nucleotide location in the MSA. All 7708 positions are plotted (figure 3), but most of the values are small and cluster around zero. The larger coefficients (which contribute strongly to the PCA) can be seen in figure 3 and there were three clearly distinct patterns of nucleotide position contributions (figure 3), which corresponded to three major clades seen in the phylogenetic tree of type 1 EBV strains [6]. Europe, Africa and USA strains were in the same major clade of the phylogenetic tree [6] but then separated within that clade. Indonesia and Papua New Guinea were in another major clade of the phylogenetic tree and have similar patterns in figure 3. Asian strains were in a separate clade and have a unique pattern in figure 3. By choosing a unique region from the larger coefficients of each pattern, we obtained lists of MSA positions that distinguished these geographical regions (electronic supplementary material, table S3). The full geographical separations in the PCA involve many small contributions distributed all along the genome (figure 1*c*), but the selected positions were sufficient to characterize an EBV genome into one of these groups. Studies on EBV transmission will require SNPs that can distinguish EBV strains and this type of analysis can identify such strain markers. SNPs in *LMP1* and *EBNA3* genes were prominent in some of these lists but not in others, and many other gene regions were represented (electronic supplementary material, table S3); these will facilitate future studies that require markers of EBV strains.

In this analysis we have not distinguished between EBV sequences from cancer cases and normal infections (details listed in [6]) because it appears that the type 1/type 2 and geographical differences are much larger. This is illustrated in a PCA plot in which contributions are coloured red for cancer or green for normal (electronic supplementary material, figure S3). There is no systematic difference in the patterns at this level of analysis, so interpreting the patterns of variation we have observed in this paper in type 1 EBV strains as geographical is not confounded by the mixture of cancer and normal strains present in the dataset.

### (d) Repeat arrays and use of viral genetics to determine functional significance in the IR-1 repeat

**Figure 4.**
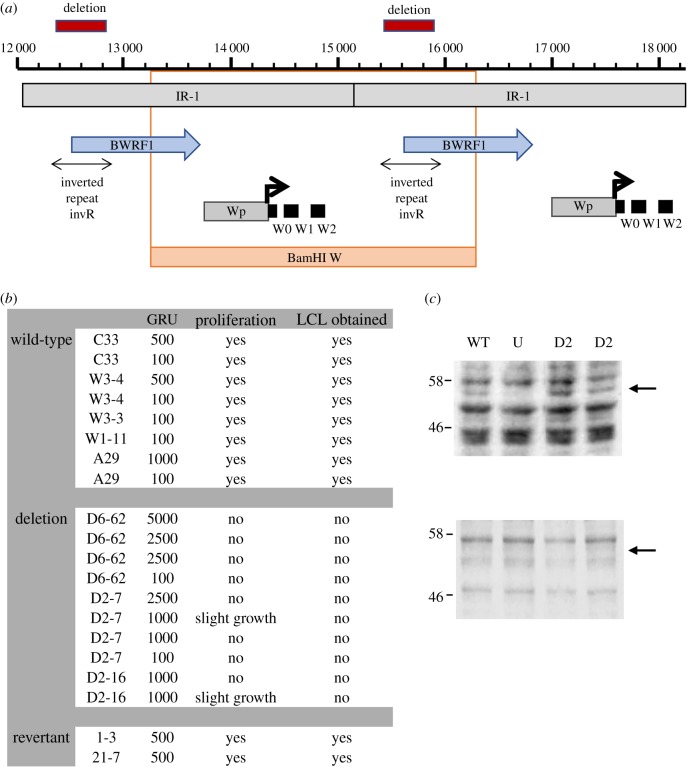
(*a*) Diagram of two IR-1 repeat units, numbered according to the NC007605 reference EBV sequence, showing the positions of the deletions of the inverted repeat invR. The locations of the BWRF1 open reading frame and the known Wp promoter region are marked, together with the W0, W1 and W2 exons of RNA expressed from Wp. The relationship between IR-1 and the repeated BamHI W restriction fragment is also indicated. (*b*) Transformation assay for human B lymphocytes using wild-type, revertant or invR deletion mutant virus strains. C33, W3-4, W1-11 and A29 are different producer lines for the wild-type virus and D6-62, D2-7 and D2-16 are producer lines for the invR-deleted virus. 1-3 and 21-7 are revertant virus strains. Virus titre is the quantity of virus (in green Raji units—GRU) added to 10^6^ B cells and the outcome is scored as cell proliferation observed under the microscope 2 days after infection or lymphoblastoid cell line (LCL) outgrowth two weeks after infection. (*c*) Upper panel: EBNA-LP western blot showing endogenous protein in Raji cells and the extra EBNA-LP (arrowed) from superinfecting virus 2 days after infection with wild-type (WT) or invR (D2) virus. Uninfected Raji cells (U) do not have that band. Positions of size markers 46 kD and 58 kD are indicated. Lower panel: Same as upper panel but assayed 2 h after addition of virus, showing that the extra band is absent so was not owing to protein present in the virus preparation but a consequence of viral gene expression in the super-infected cells.

Because the size of repeat units within several of the EBV repeat arrays is greater than paired Illumina sequence read lengths, it is difficult to assign individual reads to any individual copy of the repeat. This limits the analysis of the large repeat arrays in the EBV genome. Mutating all the copies of a repeat array is also challenging with the conventional BAC genetics that is used in herpesvirus research. Small variations in the sequence of individual repeat copies can allow deconvolution of the reads and construction of parts of the repeat array. We did this successfully for the IR-1 major internal repeat [3]. The IR-1 region (figure 4*a*) is important because it encodes the Wp promoter for initial expression of the EBNA mRNAs upon infection. In addition to some of the exons for EBNA-LP, a long open reading frame (BWRF1) is present in the IR-1 sequence (figure 4*a*); BWRF1 was found to be conserved (or to be even longer than in the reference NC007605) in most EBV strains [3].

No protein product has yet been identified for BWRF1 and part of that region of the IR-1 nucleotide sequence includes a large inverted repeat [18] known as the invR structure (figure 4*a*). As a first step to determining if these genetic features play a functional role in EBV, we constructed a mutated EBV strain in which all seven copies of the invR sequence are deleted from the B95-8 IR-1 repeat array. The invR deletion also removes the 5′ end of BWRF1 (figure 4*a*). The mutagenesis was done using a novel approach to BAC genetic engineering we described recently [8] and a revertant strain was also made. Infection of primary human B cells with several clones of this invR-deleted virus was compared with the prototype B95-8 BAC virus and revertant virus; outgrowth of EBV-transformed lymphoblastoid cells was measured. The deletion completely blocked B cell transformation (figure 4*b*) but the revertant virus was similar to the prototype virus, indicating the phenotype was owing to the deletion of the invR sequence. To test whether the deletion simply inactivated gene expression from Wp, we also superinfected Raji cells with the prototype or the invR deletion virus. Expression of EBNA-LP from the infecting virus (its EBNA-LP has a different size on the SDS gel from the endogenous EBNA-LP in Raji cells) was similar with both wild-type and invR deletion strains, indicating that Wp is still functional and that the RNA splicing events required to make EBNA-LP protein are not prevented by the invR deletion.

## 4. Discussion

Known viral genes, origins of replication and gene expression control regions explain most of the EBV genome but there are still a few sections of completely unknown function. One of these regions is the inverted repeat sequence (invR) within the IR-1 major internal repeat array. Deleting this invR sequence abolished the ability of the virus to transform human B cells into lymphoblastoid cell lines. The invR deletion did not affect the expression of EBNA-LP from the incoming viral genome in superinfected Raji cells, so it appears that Wp function and the splicing of EBNA-LP RNAs are not compromised in those cells. The deletion removes part of the BWRF1 open reading frame and the intron containing invR has also been reported to produce a stable RNA (ebv-sisRNA-2) in the nucleus [19]. This ebv-sisRNA-2 has recently been found to bind several cell regulatory proteins [20], so it is possible that our invR mutation affects cell transformation through that type mechanism. Also, it is not currently known whether BWRF1 is actually expressed as a protein in EBV-infected cells; no antibody for the protein is available and a survey of stable mRNAs from that region of the EBV genome did not show abundant RNA in the BWRF1 region in LCLs [2]. The strong effect of the new mutation we have identified here will, however, justify further investigation of BWRF1 expression and the role of the invR sequence. The time course of expression of the EBNA RNAs during infection approximately follows their distance from the Wp promoter, so its proximity to Wp suggests that it may be important to look very early after infection for BWRF1 expression.

Using a combination of PCA and SNP counting, we identified some regions of the genome that contribute to geographical variation (figure 3, electronic supplementary material, figure S2, tables S2 and S3 and ref. [6]). There are both distributed local sequence variations and localized differences in repeat copy numbers or indels at several locations. The SNP counting approach that we used identifies all of these, but the visualization using the accumulated SNPs in a sliding window tends to emphasize the indels, since our approach characterizes each nucleotide gap as an independent SNP. Choosing SNPs with high eigenvector coefficients that clustered in the PCA plots (figure 3) allowed identification of localized SNPs (electronic supplementary material, table S3), which will be useful as strain markers in future studies of EBV transmission. Combinations of the indels that we focused on are sufficient to discriminate between the geographical regions, but the more distributed local sequence variations also play an important role in distinguishing the variants.

The incidence of EBV-associated diseases varies greatly in different parts of the world [1]. The selective presence of the viral genome in these diseases offers great opportunities for targeted therapy and early detection of disease. Immunization against EBV is likely to be able to prevent infectious mononucleosis [21] and EBV-based immunotherapies are being investigated for some types of cancer. Understanding the natural variation in EBV genome sequences is important because this provides a background upon which mutations or sequence differences in endemic strains can be identified that may be relevant to the diseases, diagnostic techniques or potential therapies. There is already some evidence for mutations in EBV being linked to certain types of cancer. For example, about 10% of African BL cases contain EBV with a deletion of the EBNA2 locus, which has been proposed to result in a higher expression of the EBV BHRF1 homologue of the anti-apoptosis BCL2 protein [22]. Also, mutations in the *EBNA3B* gene have been shown to make EBV more oncogenic, producing disease similar to diffuse large B cell lymphoma (DLBCL) in a mouse model system and EBNA3B mutations have been found in human DLBCL, BL and Hodgkin's lymphoma cases [23]. The V3 variant of the EBV Zp promoter (which contains an additional NFAT binding site) causes increased expression of BZLF1 and the EBV lytic replication cycle [24]. The V3 Zp is enriched in cases of African BL [24] and Chinese and Indonesian NPC [6], suggesting a role for enhanced viral replication in development of those diseases. Because of the different geographical incidences of EBV-associated diseases, it is important to understand the geographical variation of EBV to provide a context for additional identification of cancer risk alleles.

Our collection of 241 EBV genome sequences (with 217 type 1 EBV sequences) includes samples from many diseases and from healthy people from different parts of the world [6] but their distribution is unbalanced, so it is limited in its ability to correlate variation with disease. It is, however, very useful as a first step in identifying the variation that exists and will provide the basis for more targeted studies focused on specific diseases, and the functional diversity of viral proteins.

## Supplementary Material

Fig S1

## Supplementary Material

Fig S2

## Supplementary Material

Fig S3

## Supplementary Material

Table S1

## Supplementary Material

Table S2

## Supplementary Material

Table S3

## Data accessibility

EBV genome sequences are in GenBank, accession numbers are in [6] and the MSA is in electronic supplementary material, figure S1.
