# Supplementary figures and images for: Essential role of inverted repeat in Epstein–Barr virus IR-1 in B cell transformation; geographical variation of the viral genome
Source: Philos Trans R Soc Lond B Biol Sci. 2019 Apr 8;374(1773):20180299. doi: 10.1098/rstb.2018.0299 (PMC6501908; doi:10.1098/rstb.2018.0299)

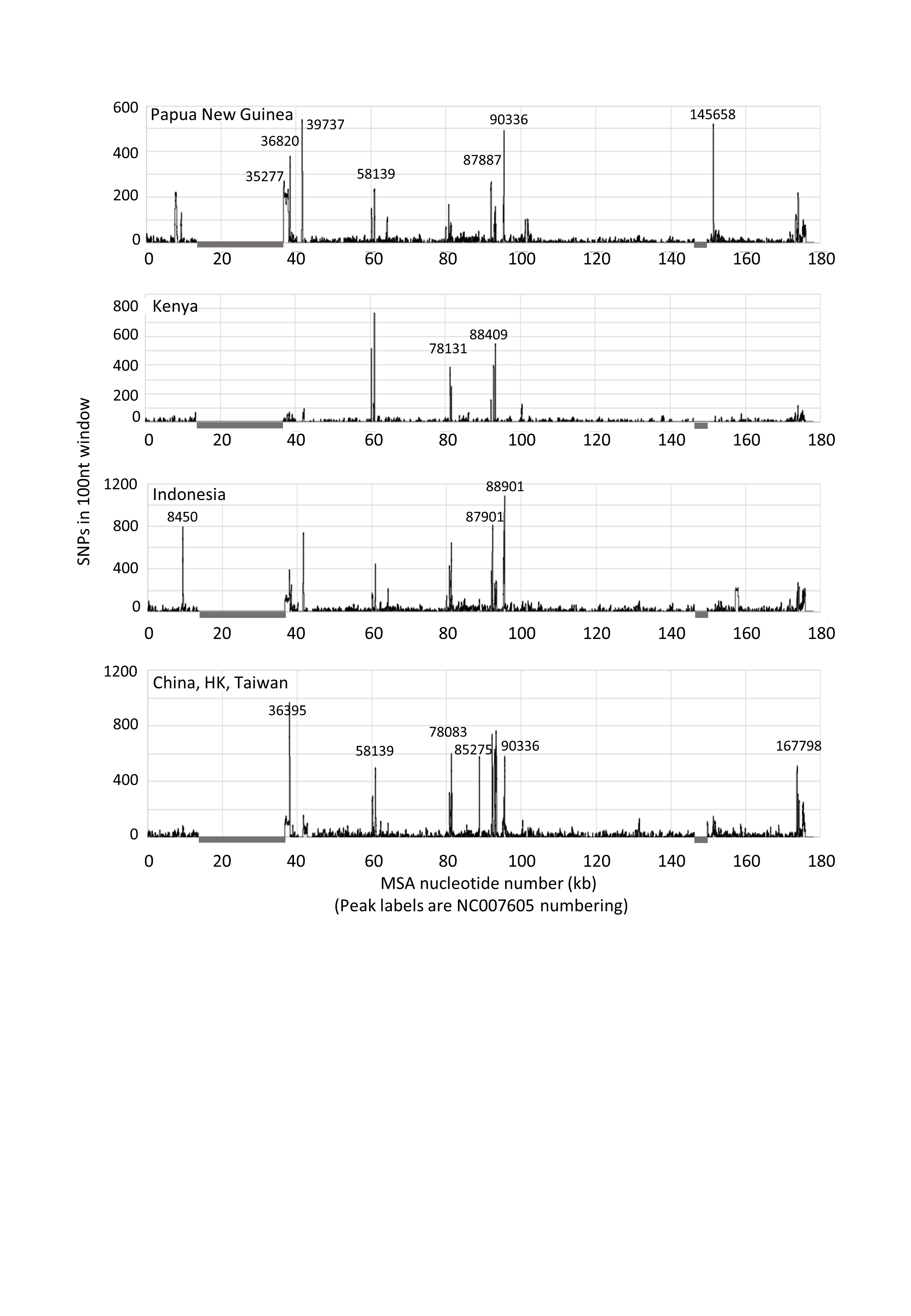

Supplement: Fig S2 [file rstb20180299supp2.tif]

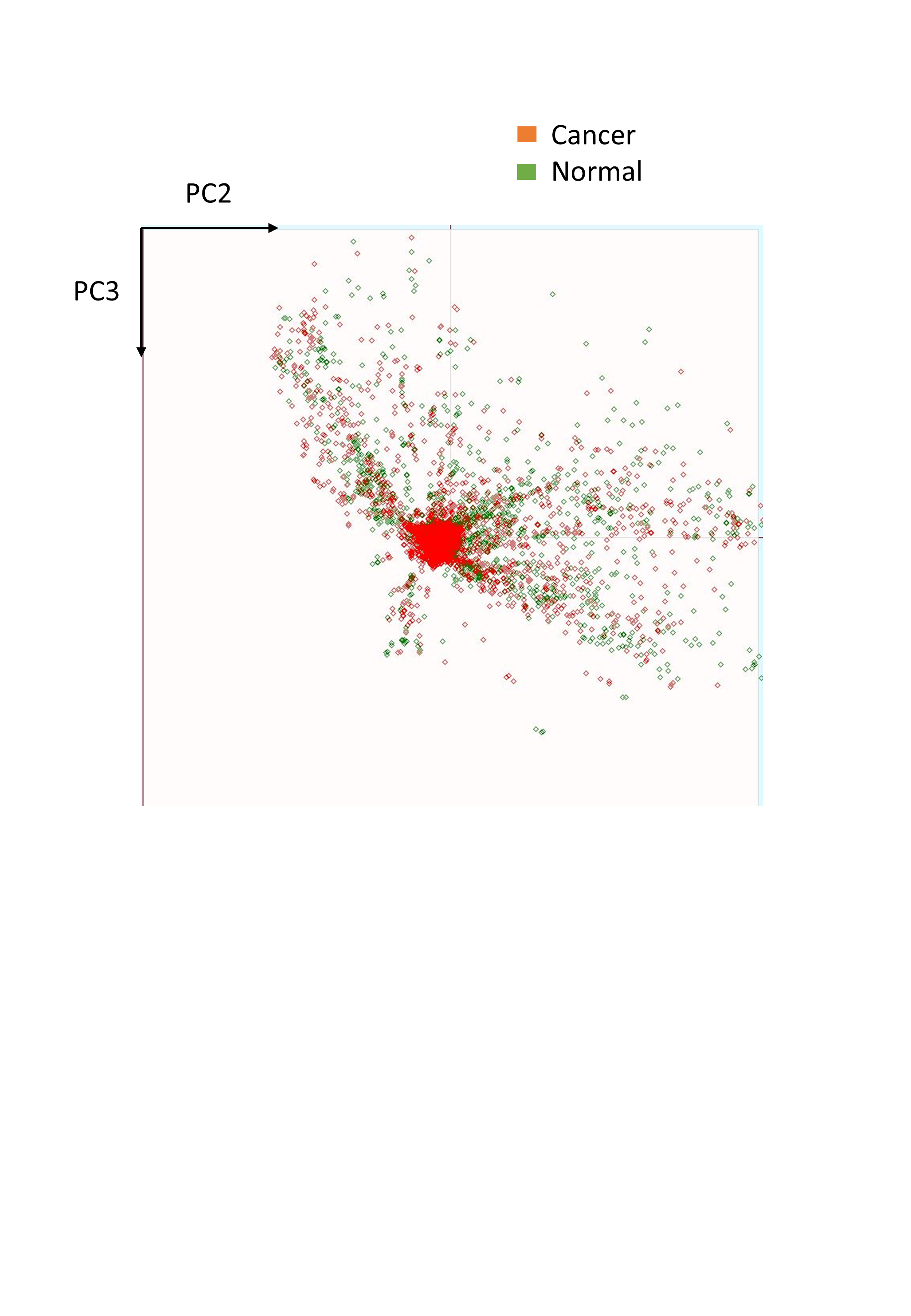

Supplement: Fig S3 [file rstb20180299supp3.tif]
